# Pioneer of burn medicine in China: Professor Li Ao and “Li Ao spirit”

**DOI:** 10.1007/s13238-020-00703-z

**Published:** 2020-03-14

**Authors:** Yaling Liao, Quaming Zou, Jiang Gu

**Affiliations:** National Engineering Research Center of Immunological Products, Department of Microbiology and Biochemical Pharmacy, College of Pharmacy, Third Military Medical University, The 30th, Gaotanyan Street, Shapingba District, Chongqing, 400038 China

The 2019 marks twenty years since Professor Ao Li (黎鳌) passed away. Although he has left us, the “Li Ao Spirit” has lived on and continuously encourages us to climb higher in scientific research and make more contributions to our people and country (Fig. 1).

**Figure 1:**
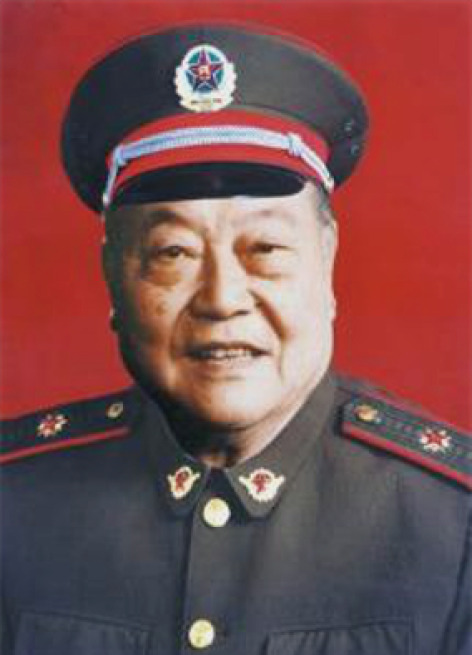
**Professor Ao Li**

Professor Li is a well-known Chinese expert in burn surgery and a member of the Chinese Academy of Engineering. Mr. Li, also named Shengxu Li (黎升旭), was born in Changsha, Hunan Province in May 1917. He graduated from the National Shanghai Medical College in 1941, and had a long and distinguished career in medicine. His various positions over the years included Associate Professor at the National Chung Cheng Medical College, Professor at the Third Military Medical University, Director of the Burn Research Institute of the Third Military Medical University, and the Vice President of the Third Military Medical University. As one of the pioneers and founders of burn medicine in China, professor Li led comprehensive multidisciplinary research on the pathogenesis and prevention of inhalation injury. For his great contribution to the field of burn research, he was not only the recipient of the Everett Idris Evans Memorial Lecture Award of the American Burn Association in 1994, but also won first prize in both the Chinese Science and Technology Advancement Award and the Military Scientific and Technological Progress Award among other accolades.

## Li Ao Spirit: Selflessness, Patriotism and Devotion

After graduating from high school, Mr. Li was recommended to study in the Department of Physics or Mathematics at Nanjing Central University. However, touched by the sufferings of the general public caused by a lack of medical knowledge and medicine and his father’s illness, Mr. Li was determined to become a doctor. In 1935, he was given the opportunity to study at Shanghai Medical College, where he started his medical career. After graduating, he successively presided over general surgical work in the National Chung Cheng Medical College, the Third Military Medical University and other institutions, saving thousands of lives.

In the late 1950s, the number of burn patients increased drastically due to the country’s efforts in increasing steel production, but there were no effective therapies available at that time (Li et al., 1983). In order to solve this urgent problem, Professor Li adjusted the direction of his research and began to investigate the mechanism of burns. He gathered the resources of the whole school (the Third Military Medical University) and achieved many advances in this field. Together with his team, Professor Li significantly improved the recovery rate of burn patients and made a great contribution to protecting people’s health.

## Li Ao Spirit: Hard-Working Entrepreneurship

**Figure 2:**
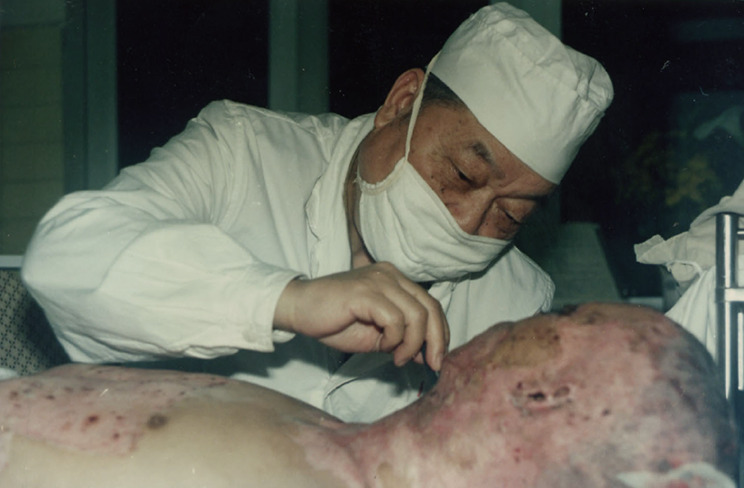
**Professor Ao Li was examing the change of burn patiens for first-hand observations**

In 1958, Professor Li began to build a burn ward, starting with only six beds and four doctors. From then on, he devoted all his time and energy to the research of burns. He stayed at the bedsides of burn patients day and night for first-hand observations (Fig. 2). Moreover, in 1963, by collaborating with the other researchers in the Third Military Medical University, he successfully developed a set of novel and effective burn treatment programs. For example, he first put forward the idea that when transportation conditions are poor, patients with large-scale burns should not be transported over long distances due to the shock incurred. Instead, anti-shock treatment should be timely applied. In addition, he identified the type of bacterial pathogens and the of infections post burns after a large number of investigations. Moreover, he simplified the cumbersome isolation measures for the sake of earlier debridement. He also conducted measurements of the body surface area of Chinese people, and proposed China-specific formulations for the calculation of the burn area. On the basis of a large number of cases, he summarized the formula of fluid replacement for burn patients during the shock period (Li, 2001).

These measures greatly reversed the passive situation in treating patients with large-scale burns at that time. Professor Li’s concepts and methods of burn treatment with Chinese characteristics were promptly accepted and promoted by his peers, laying a solid foundation for the development of burn medicine in China.

## Li Ao Spirit: Embracing Challenges

The high mortality rate of inhalation injury is a serious problem in burn treatment, and there was no systematic study focusing on this issue worldwide at that time for its complex pathogenesis. Despite his awareness of the difficulties, Professor Li chose inhalation injury as his main research direction. He organized nearly 100 researchers from 16 departments of the Third Military Medical University, and set up a burn collaboration group to focus on inhalation injury research. The research group achieved many important progressions.

For instance, this research project clarified the pathological morphology and pathophysiology of inhalation injury, revealed its pathogenesis and led to a set of early treatment measurements which reduced complications and improved the recovery rate of burn patients. These research progressions and treatment experiences were included in his monograph *Inhalation Injury* (《吸入性损伤》), which was published by People’s Military Medical Press in 1993 (Li et al., 1993). The fruits of this research project won the 2nd Prize of Chinese Science and Technology Advancement Award.

In the 1980s, Professor Li found that severe shock, ischemia, hypoxia and subsequent uncontrolled inflammatory reactions are the main causes of internal organ damage in burn patients. To further investigate the mechanism, he and Professor Jixiang Shi (史济湘) launched the research project named “Study on the Pathogenesis of Early Burn Injury and Wound Healing Mechanism”. The project was sponsored by the National Natural Science Foundation. After five years of hard work, they discovered that macrophages act as an initiator in the uncontrolled inflammatory response after burns, verified that vascular endothelium played the central role in the pathogenesis of early organ damage, clarified the mechanism of the early intestinal damage and intestinal infections, and proposed corresponding treatment measures (Huang et al., 2001). These findings were highly recognized by international burn researchers and made Professor Li the recipient of the Everett Idris Evans Memorial Lecture Award of the American Burn Association in 1994.

## Li Ao Spirit: Cultivating Young Talents

**Figure 3:**
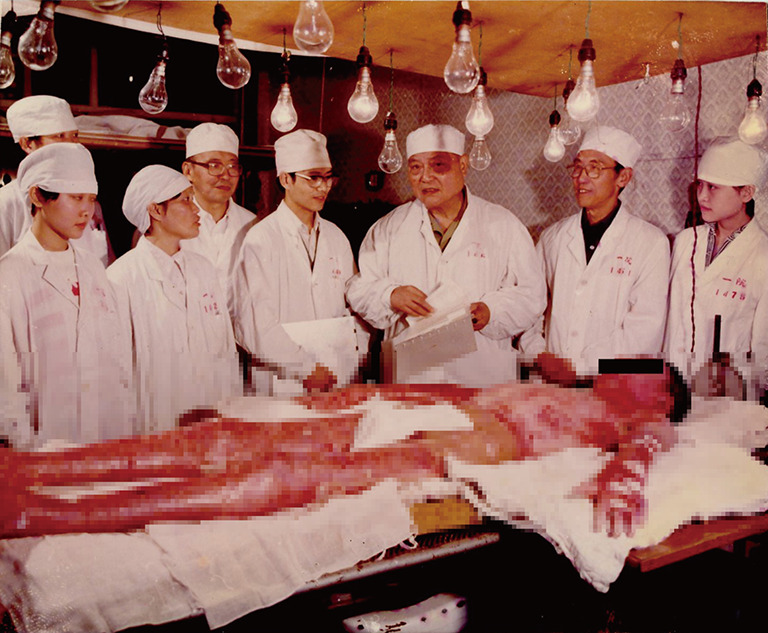
**Professor Ao Li was educating students beside the bed**

Professor Li attached great importance to the growth and cultivation of young scientists. He emphasized training in practice, encouraged young scientists to participate in clinical operation, classroom teaching and laboratory tasks (Fig. 3). He provided a stage for the young generation to showcase their talents and helped them grow into principle academic leaders.

In 1986, Professor Li won the National Science and Technology Progress Award. He donated the bonus of the award to establish the Outstanding Paper Award in Burn Medicine for Young Army Scientists to encourage young scientists and technicians to engage in burn research. In 1996, Professor Li won the China Engineering Science and Technology Award and Military Technical Major Contribution Award. Again, he donated the bonus of both awards, a total of 150,000 Chinese Yuan, to set up the Li Ao Burn Medicine Fund so as to reward young and middle-aged scientists who contributed to the development of burn medicine. Ten of the recipients of the Li Ao Burn Medicine Fund have become the leading scientists of burn medicine in China. In 1996, Professor Li was awarded the title of “Outstanding Mentor” by the General Logistics Department of Chinese People’s Liberation Army.

Professor Li has devoted his whole life to the cause of burn medicine in China and made a lot of remarkable achievements. Although he left us twenty years ago, we are still always encouraged and inspired by the “Li Ao Spirit” of selflessness, diligence and entrepreneurship. With the great efforts of generations of doctors and scientists, we believe burn medicine research in China will bring about more and more breakthroughs and play a leading role in the international arena.

## References

[CR1] Huang YS, Yang ZZ et al (2001) Clinical analysis of measures for preventing early postburn damage in improving survival rate of burn patients. J Third Mil Med Univ 23(02):217–220 (黄跃生, 杨宗城, 肖光夏, 汪仕良, 黎鳌 (2001). 烧伤早期损害的防治措施对提高烧伤存活率的作用, 第三军医大学学报, 23(02):217–220)

[CR2] Li Z, Wang JH et al (1983) Analysis of mortality rate of 3617 burned patients. J Third Mil Med Univ 5(03):182–190 (李照洲, 王甲汉, 朱佩芳, 黎鳌, 尹全焕 (1983) 3617例烧伤病人病死率分析, 第三军医大学学报, 5(03):182–190)

[CR4] Li A (2001) The past, present and future of burn treatment research in China. Chin J Burns 17(01):5–7 (黎鳌 (2001). 我国烧伤救治研究的过去, 现在和未来, 中华烧伤杂志, 17(01):5–7)

[CR3] Li A, Yang ZZ (1993) Inhalation Injury. People’s Military Medical Press. ISBN: 7-80020-363-8 (黎鳌, 杨宗城 人民军医出版社, 吸入性损伤, ISBN: 7-80020-363-8)

